# Torque Teno Virus (TTV) Plasma Load and Immune Reconstitution in People Living with HIV: A Systematic Review

**DOI:** 10.3390/microorganisms14061386

**Published:** 2026-06-22

**Authors:** Federico Cesanelli, Ottavia Nozza, Martina Salvi, Maria Alberti, Irene Scarvaglieri, Giorgio Tiecco, Francesca Mosti, Maria Antonia De Francesco, Eugenia Quiros-Roldan

**Affiliations:** 1 Department of Clinical and Experimental Sciences, Unit of Infectious and Tropical Diseases, University of Brescia and ASST Spedali Civili di Brescia, 25123 Brescia, Italy; f.cesanelli@unibs.it (F.C.); o.nozza@unibs.it (O.N.); m.salvi026@unibs.it (M.S.); m.alberti035@studenti.unibs.it (M.A.); i.scarvaglieri@unibs.it (I.S.); g.tiecco@unibs.it (G.T.); f.mosti@unibs.it (F.M.); 2 Department of Molecular and Translational Medicine, University of Brescia, 25123 Brescia, Italy; maria.defrancesco@unibs.it; 3 Highly Specialized Laboratory, ASST Spedali Civili of Brescia, 25123 Brescia, Italy; 4 Department of Global Public Health, Karolinska Institute, 17177 Stockholm, Sweden

**Keywords:** TTV, torque teno virus, HIV

## Abstract

Background: Torque teno virus (TTV) is a ubiquitous, non-pathogenic component of the human virome whose role in people living with HIV (PLWH), particularly during antiretroviral therapy (ART)-mediated immune reconstitution, remains unclear. This systematic review aimed to synthesize available evidence on TTV viral load in PLWH, focusing on its relationship with immunological markers. Methods: This systematic review followed the Preferred Reporting Items for Systematic Reviews and Meta-Analyses (PRISMA) 2020 guidelines. A comprehensive literature search was conducted in MEDLINE, Web of Science, and Scopus in January 2026 to identify studies assessing plasma TTV viral load before and/or during ART and reporting immunological outcomes. Eligible studies included prospective and retrospective longitudinal studies, cross-sectional studies, and mixed designs assessing plasma TTV viral load in relation to ART status and immune recovery markers. Results: Thirteen studies (n = 1700 PLWH) were included, predominantly observational and conducted in adult populations. Most studies (76.9%) reported a significant inverse association between TTV viral load and CD4 T-cell count, while all studies assessing HIV viral load found a direct correlation with TTV levels. An inverse relationship with the CD4/CD8 ratio was consistently observed where evaluated. Higher TTV loads were reported in ART-naïve individuals and in those with advanced immunosuppression, with longitudinal studies indicating a general decline during ART. Overall, methodological heterogeneity and moderate risk of bias were common. Conclusions: TTV viral load shows a consistent inverse association with CD4 cell count and may reflect global immune dysfunction in PLWH beyond conventional markers. However, its clinical utility remains investigational due to the heterogeneity in the study design, limited data on longitudinal dynamics, and lack of standardized assays and thresholds.

## 1. Introduction

Torque teno virus (TTV) is a ubiquitous, non-enveloped, circular single-stranded DNA virus belonging to the *Anelloviridae* family, a highly diverse group currently comprising more than 30 genera and over 150 species [1]. Human infection is primarily associated with three genera *Alphatorquetenovirus* (including TTV), *Betatorquetenovirus* (Torque teno mini virus, TTMV), and *Gammatorquetenovirus* (Torque teno midi virus, TTMDV), which together represent a major component of the human virome [2]. TTV displays remarkable genetic heterogeneity, with at least five major phylogenetic groups differing by approximately 50% of their genomic sequence and multiple genotypes within each group [3]. This extensive variability reflects its long-standing co-evolution with humans and contributes to its widespread global distribution [4]. TTV is typically acquired early in life through multiple transmission routes, including breast milk, saliva, respiratory droplets, and fecal–oral exposure [5,6]. As a result, its prevalence reaches 65–94% in adulthood [7]. The virus shows a particular tropism for lymphocytes, although it can be detected in a broad range of tissues and body fluids, supporting its classification as a pantropic virus [8,9]. Importantly, despite its persistent presence in human host, TTV has not been linked to any specific human disease and is generally regarded as a non-pathogenic commensal virus [10,11]. Similarly, other human anelloviruses, including TTMV and TTMDV, may establish persistent infections. However, within the setting of HIV infection, available evidence has mainly focused on TTV, while data on other anelloviruses remain comparatively limited.

A defining feature of TTV biology is the strong dependence of its replication on the host immune competence [12,13]. In immunocompetent individuals, viral replication is tightly controlled, whereas in conditions of immune suppression, such as after solid organ transplantation, during chemotherapy, or in people living with HIV (PLWH) with an advanced infection, TTV plasma loads rise substantially [13,14]. This inverse relationship between immune function and TTV viremia has led to growing interest in TTV as a surrogate biomarker of global immune status [13,15]. In PLWH, established markers such as CD4 T cell counts, the CD4/CD8 ratio, and plasma HIV RNA remain the cornerstone of clinical monitoring. However, these markers provide information on immune reconstitution and virological control, while they may not fully reflect residual immune dysregulation or the functional quality of immune recovery, particularly in individuals with sustained virological suppression [16,17]. For this reason, TTV DNAemia has been proposed as a potential complementary marker, rather than an alternative to current HIV-related parameters, because it may reflect the overall balance between viral replication and host immune surveillance [18].

In this context, TTV has attracted increasing attention because higher viral loads appear to indicate reduced immune control rather than direct immunosuppressive activity [18,19]. Nevertheless, its role in the setting of HIV infection remains incompletely elucidated. In particular, the dynamics of TTV plasma viral load during antiretroviral therapy (ART)-induced immune reconstitution, as well as its association with clinical and immunological outcomes in PLWH, are not yet fully characterized [20]. This systematic review aims to synthesize the current body of evidence regarding TTV viral load in PLWH, with particular emphasis on its relationship with established immunological markers and its potential role as complementary biomarker of immune status.

## 2. Methods

This systematic review followed the Preferred Reporting Items for Systematic Reviews and Meta-Analyses (PRISMA) 2020 guidelines (Supplementary Materials) [21]. The review protocol was registered in the PROSPERO database (Registration ID: CRD420261300212) on 5 February 2026.

### 2.1. Eligibility Criteria

Eligible studies included adult or pediatric PLWH receiving stable ART in whom plasma TTV viral load was assessed before and/or during antiretroviral therapy, with ART exposure clearly described and correlated to immunological parameters relevant to immune recovery. Prospective longitudinal cohort studies, prospective clinical trials, retrospective longitudinal analyses, retrospective observational studies, cross-sectional investigations, and mixed designs combining retrospective and prospective elements were considered if they evaluated TTV load kinetics in relation to ART status, ART exposure, or ART-associated immune recovery. Studies were excluded if they did not include PLWH, did not report plasma TTV load measurements, failed to evaluate immune reconstitution using standardized immunological markers, or were reviews, case reports, editorials, conference abstracts, or animal studies.

### 2.2. Information Sources and Search Strategy

A comprehensive literature search was conducted in PubMed/MEDLINE, Web of Science and Scopus in January 2026 using the following search string: ((TTV OR Torque Teno Virus) AND HIV). No time restrictions were applied. Only published studies available in English were considered. In addition, the reference lists of all included articles were screened to identify any additional relevant studies.

### 2.3. Selection and Data Collection Process

A team of two resident doctors in Infectious and Tropical Diseases of the University of Brescia, Italy, independently screened the abstracts of all retrieved records and selected potential eligible articles according to the established inclusion and exclusion criteria (ON and MS). A Professor in Infectious and Tropical Diseases of the University of Brescia, Italy (EQR) and a Professor in Microbiology of the University of Brescia (MADF) revised the included and the rejected papers. Then, the selected papers were equally distributed among each resident doctor to assess full-text eligibility and perform data extraction. Each resident doctor read the assigned articles and collected and synthesized the relevant data using a detailed database. Afterwards, across-checking phase was performed: each reviewer re-examined data extracted by a colleague to ensure consistency and accuracy. Disagreements were resolved by a joint discussion supervised by the Professor in Infectious and Tropical Diseases (EQR) and the Professor in Microbiology (MADF). Reviewers were not blinded to study authors, journals, or study outcomes during study selection or data extraction.

### 2.4. Data Items

For each included study, data were systematically extracted by independent reviewers (ON, MS, MA, FM, and IS) using a predefined structured extraction form developed by the review team in accordance with JBI guidance and adjusted for the objectives of this systematic review. Extracted information included study characteristics (first author, year of publication, country, journal, and study design) and population details such as sample size, age, sex, ART status and duration, HIV viral load, CD4 T-cell count, CD8 T cell count, and CD4/CD8 ratio, when available. Clinical and virological data included the timing of sample collection, biological matrix, methodological approaches used for TTV detection or quantification, and TTV viral load at baseline and, when available, at follow-up time points during immune reconstitution. TTV measurements were extracted as reported by the original studies. The primary outcome of interest was the correlation between TTV plasma load and immune reconstitution, assessed through immunological markers, particularly absolute T-cell counts and the CD4/CD8 ratio. When available, TTV viral load and immunological parameters were extracted at matching time points. Missing or unclear information was recorded as “not available” (NA).

### 2.5. Synthesis Methods

Considering the heterogeneity in study design, populations, ART exposure, biological specimens, and TTV quantification methods, a quantitative meta-analysis was not considered appropriate. Therefore, a qualitative synthesis was performed. Extracted data were summarized descriptively across studies, focusing on key domains: study characteristics (design, sample size, population), patient demographics, ART exposure, laboratory methods (TTV quantification techniques and specimen types), and immunovirological parameters. Continuous variables, including TTV viral load, were reported as medians and ranges when available, while categorical variables were expressed as counts and percentages. The primary objective of the synthesis was to evaluate the association between TTV viral load and immunological markers, particularly CD4 cell count. Reported associations were categorized as inverse, direct, or absent based on the direction and statistical significance of correlations provided in individual studies. Findings were synthesized narratively, highlighting consistency or discrepancies across studies.

### 2.6. Bias and Certainty Assessment

The methodological quality of the included studies was assessed using the Joanna Briggs Institute (JBI) Critical Appraisal Checklists for analytical cross-sectional and cohort studies, as appropriate to each study design [22]. Each study was independently evaluated across the following domains: clarity of inclusion criteria, validity and reliability of exposure measurement, validity and reliability of outcome measurement, identification of confounding factors, strategies to address confounding, appropriateness of statistical analysis, and adequacy of study population and setting description. Each item was rated as “Yes”, “No”, or “Unclear”. An overall risk of bias judgment (low, moderate, or high) was assigned to each study based on the number and relevance of unmet criteria. The results are represented as traffic light and weighted bar graphs generated by using the generic dataset model of the Risk of Bias Visualization (ROBVIS) package [23].

## 3. Results

### 3.1. Study Selection and Search Results

**Figure 1:**
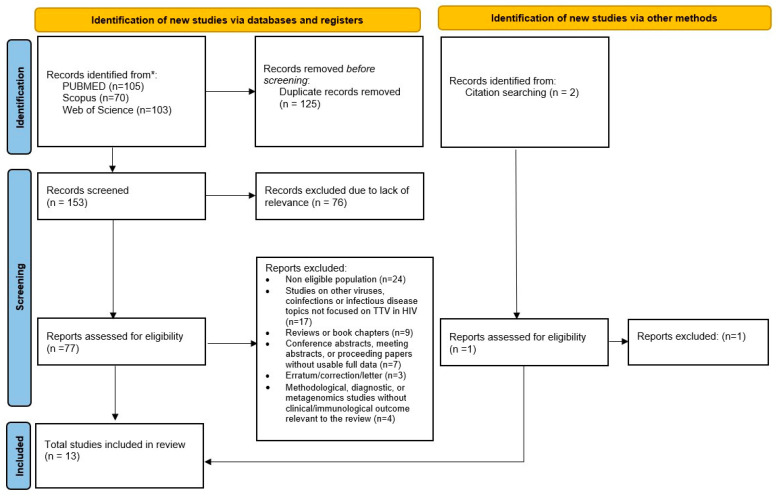
PRISMA 2020 flow diagram for new systematic reviews.

A total of 278 records were retrieved from MEDLINE, Scopus, and Web of Science and two additional records were identified through backward citation searching. A total of 125 duplicate records were removed. After title and abstract screening of 153 records, 76 were excluded due to lack of relevance. The remaining records underwent full-text assessment: 77 reports were evaluated for eligibility from database searching, and one report from citation searching. Among these, 65 studies were excluded because they did not provide sufficient data for extraction. Ultimately, 13 studies met the inclusion criteria and were incorporated into the qualitative synthesis. The study selection process is illustrated in the flow diagram (Figure 1), while the characteristics of the included studies are summarized in Table 1 [18,19,20,24,25,26,27,28,29,30,31,32,33].

**Table 1:** Characteristics of included studies [18,19,20,24,25,26,27,28,29,30,31,32,33].

First Author, Year	Study Design	Sample Size	Age, Median/Range	ART Status/Duration	Specimen/Biological Matrix	TTV Method	TTV Measure Reported	Role in Synthesis
Tarancon-Diez, 2024 [18]	Retrospective	57	17 years (14–20.5)	65 months	Plasma	TTV R-Gene^®^ kit, bioMérieux	3.19 log10 copies/mL	Main circulating blood-based synthesis
Lapa, 2021 [24]	Retrospective	63 HIV/HCV coinfected	53 years (49–56)	NA	Plasma	TTV R-Gene^®^ kit, bioMérieux	2.89 log10 copies/mL	Main circulating blood-based synthesis
Honorato, 2022 [25]	Cross-sectional	276	44.8 years	4.0 ± 0.9 years	Saliva	In-house RT-PCR	3.3 log10 copies/mL in males; 2.4 log10 copies/mL in females	Salivary/mucosal evidence; not included in main plasma-based synthesis
Esser, 2024 [19]	Retrospective	186	42.7 years	ART-naïve	Plasma	In-house RT-PCR	7.33 log10 copies/mL	Main circulating blood-based synthesis
Devalle, 2009 [26]	Retrospective	15	43.9 years	22.1 ± 5.4 months	Plasma	In-house RT-PCR	5.89 log10 copies/mL	Main circulating blood-based synthesis
Elesinnla, 2020 [27]	Cross-sectional	130	NA (22–74)	NA	Serum	In-house RT-PCR	NA	Qualitative serum-based evidence; not included in quantitative range because viral load was not extractable
Thom, 2007 [28]	Cross-sectional	19; 13 with AIDS, 6 with pre-AIDS	AIDS: 33 years (20–60); pre-AIDS: 29 years (25–31)	No ART	Bone marrow and spleen	In-house RT-PCR	7.85 log10 copies/mL in AIDS; 5.21 log10 copies/mL in pre-AIDS	Tissue-based evidence; not included in main circulating blood-based synthesis
Abbate, 2023 [20]	Longitudinal	17	39 years (33–49)	12 months	PBMCs	In-house RT-PCR	4.5 log10 copies/10^6^ PBMCs	Cellular compartment evidence; not included in main circulating blood-based synthesis
Fan, 2025 [29]	Retrospective	126; 46 on ART	47 years (40–58)	NA	Blood, LRT samples, CSF	Metagenomic NGS	TTV reported as RPM; not extractable as log10 copies/mL	Qualitative/contextual evidence; not included in main circulating blood-based synthesis
Shibayama, 2001 [30]	Cross-sectional	144	37 years	No ART	Serum	In-house RT-PCR	4.5 log10 copies/mL using UTR-target PCR; 2.6 log10 copies/mL using N22-target PCR	Main circulating blood-based synthesis
Christensen, 2000 [31]	Cross-sectional	347	NA	No ART	Serum	In-house RT-PCR	5.59 log10 copies/mL	Main circulating blood-based synthesis
Madsen, 2002 [32]	Longitudinal	15	NA	12 months	Serum	Quantitative end-point PCR	4.78 log10 copies/mL	Main circulating blood-based synthesis
Schmidt, 2021 [33]	Retrospective	301	49 years (25–92)	12 months	Plasma	In-house RT-PCR	5.36 log10 copies/mL	Main circulating blood-based synthesis

Abbreviations: RT, antiretroviral therapy; CSF, cerebrospinal fluid; HCV, hepatitis C virus; HIV, human immunodeficiency virus; LRT, lower respiratory tract; mNGS, metagenomic next-generation sequencing; NA, not available; PBMCs, peripheral blood mononuclear cells; RPM, reads per million; RT-PCR, real-time polymerase chain reaction; TTV, Torque Teno Virus. TTV values are reported as log10 copies/mL only when available and when this was the unit used in the original study.

All included studies employed observational designs, encompassing cross-sectional (38.5%), retrospective (46.2%), and prospective approaches (15.4%). The majority of studies were conducted in Europe (65%), predominantly in Italy and Germany, followed by Asia (20%), South America (10%), and Africa (5%). Most studies enrolled adult populations (92.3%), while only one [18] focused on adolescents with vertically acquired HIV. Sample sizes varied widely, ranging from small mechanistic cohorts (n = 15) to large cross-sectional analyses (n = 347).

### 3.2. Quality Assessment of the Articles

**Figure 2:**
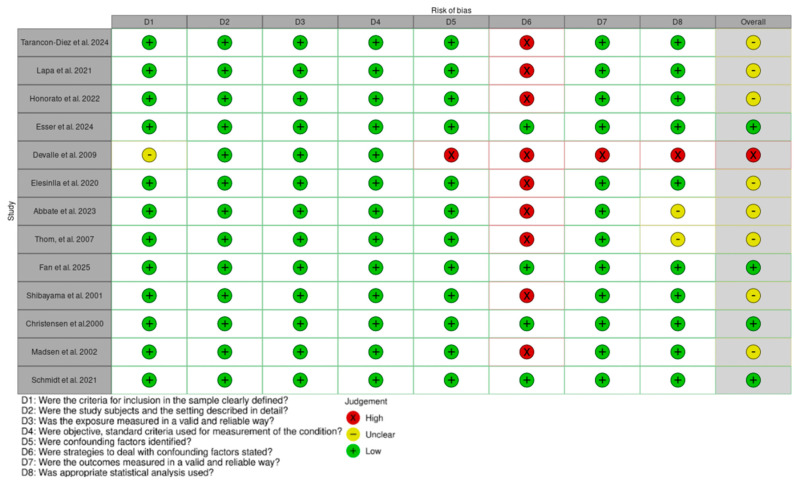
Risk of bias assessment of included studies. Traffic light plot summarizing the methodological quality of each included study according to the JBI critical appraisal tools. Each domain was rated as low risk (green), high risk (red), or unclear risk (yellow) [18,19,20,24,25,26,27,28,29,30,31,32,33].

**Figure 3:**
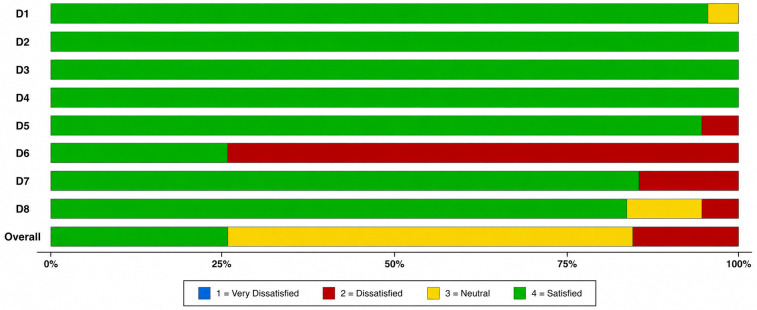
Summary of risk of bias across included studies. Bar chart showing the proportion of studies rated as low, high, or unclear risk of bias for each methodological domain. Assessments were performed using JBI tools, and results are presented as percentages of total included studies.

Overall, most studies were judged to be at moderate risk of bias (61.5%), while 30.7% were rated as low risk and the remaining 7.7% as high risk. Most studies had clearly defined research questions and appropriately described the study population and setting. In addition, methods used to quantify TTV viral load, mainly based on PCR-based techniques, were generally robust and consistent across studies (Figure 2). The main sources of potential bias were related to confounding and the strategies used to address it. In particular, 69.2% of the studies included did not adequately address confounding, either due to the lack of multivariable analyses or insufficient stratification (Figure 3). Variables such as HIV disease stage, duration and type of antiretroviral therapy, co-infections, and demographic or clinical characteristics were frequently not adjusted for, which may limit the interpretability of observed associations between TTV viral load and immune status.

### 3.3. Descriptive Analysis of the Sample

As shown in Table 2, a total of 1700 PLWH were included, predominantly male (74%), who acquired HIV infection mostly through sexual intercourse (72%). Across studies, participants were predominantly receiving ART (60%), although the specific regimens used were mostly unavailable. One study enrolled ART-naïve PLWH [19], and four investigations focused on acutely infected PLWH [20,26,32,33]. TTV quantification was most performed using in-house RT-PCR assays (69%), although some studies used commercial standardized kits or metagenomic next-generation sequencing (NGS) approaches. Biological specimens included plasma (38%), serum (31%), saliva (8%), and, in only one paper [28], lymphoid tissues such as bone marrow and spleen. The detailed study design, population characteristics, and laboratory methods extracted from each study are summarized in Table 3.

**Table 2:** Association between TTV and viro-immunological markers [18,19,20,24,25,26,27,28,29,30,31,32,33].

First Author, Year	Marker Assessed	Association with TTV	Statistical Measure Reported	*p*-Value	Interpretation
Tarancon-Diez, 2024 [18]	CD4 cell count	Inverse	r = −0.396	0.002	Higher plasma TTV was associated with lower CD4 count
Tarancon-Diez, 2024 [18]	CD8 cell count	Direct	r = 0.277	0.037	Higher plasma TTV was associated with higher CD8 count
Tarancon-Diez, 2024 [18]	CD4/CD8 ratio	Inverse	r = −0.37	0.0047	Higher plasma TTV was associated with lower CD4/CD8 ratio
Lapa, 2021 [24]	CD4 cell count	No association	NA	NA	No significant association between plasma TTV and CD4 count
Honorato, 2022 [25]	HIV viral load	Direct	Statistic not reported	<0.0001	Higher salivary TTV was associated with higher HIV viral load
Honorato, 2022 [25]	CD4 cell count	Inverse	Statistic not reported	<0.0001	Higher salivary TTV was associated with lower CD4 count
Esser, 2024 [19]	CD4 cell count	Inverse	Statistic not reported	<0.001	Higher plasma TTV was associated with lower CD4 count in ART-naïve participants
Devalle, 2009 [26]	CD4 cell count	Inverse	Statistic not reported	NA	Higher plasma TTV was associated with lower CD4 count
Elesinnla, 2020 [27]	HIV viral load	Direct	χ^2^ = 40.3	2.18 × 10^−10^	TTV detection/level was associated with HIV viral load; χ^2^ is not comparable with correlation coefficients
Elesinnla, 2020 [27]	CD4 cell count	No association	χ^2^ = 1.4	>0.05	No significant association with CD4 count
Thom, 2007 [28]	CD4 cell count	Inverse	Statistic not reported	<0.007	Tissue-based TTV was associated with lower CD4 count; not directly comparable with plasma TTV
Abbate, 2023 [20]	CD4 cell count	No association	NA	NA	No significant association with CD4 count
Abbate, 2023 [20]	CD8 central memory cells	Inverse	r = −0.408	<0.048	PBMC-associated TTV was associated with selected CD8 T-cell subsets
Abbate, 2023 [20]	CD8 effector memory cells	Direct	r = 0.59	<0.002	PBMC-associated TTV was associated with CD8 effector memory cells
Abbate, 2023 [20]	CD8^+^CD57^+^ cells	Direct	r = 0.464	0.023	PBMC-associated TTV was associated with senescent/activated CD8^+^CD57^+^ cells
Fan, 2025 [29]	CD4 cell count	Inverse	r = −0.359	<0.0061	Higher TTV abundance by mNGS was associated with lower CD4 count
Fan, 2025 [29]	CD4/CD8 ratio	Inverse	r = −0.535	<0.0001	Higher TTV abundance by mNGS was associated with lower CD4/CD8 ratio
Shibayama, 2001 [30]	CD4 cell count	Inverse	Statistic not reported	NA	Higher serum TTV was associated with lower CD4 count
Christensen, 2000 [31]	HIV viral load	Direct	Statistic not reported	NA	Higher serum TTV was associated with higher HIV viral load
Christensen, 2000 [31]	CD4 cell count	Inverse	Statistic not reported	NA	Higher serum TTV was associated with lower CD4 count
Madsen, 2002 [32]	HIV viral load	Direct	Statistic not reported	NA	TTV varied in relation to HIV viral load during follow-up
Madsen, 2002 [32]	CD4 cell count	Inverse	Statistic not reported	NA	TTV varied in relation to CD4 count during follow-up
Schmidt, 2021 [33]	CD4 cell count	Inverse	R^2^ = 0.028	0.003	Higher plasma TTV was associated with lower CD4 count, although the explained variance was small

NA, not available; TTV, Torque Teno Virus. “Direct” indicates that higher TTV values were associated with higher values of the corresponding marker; “inverse” indicates that higher TTV values were associated with lower values of the corresponding marker. Statistical measures included: r= Spearman rho correlation coefficient test; χ^2^ = chi square test; R, Pearson correlation.

**Table 3:** Principal characteristics of study design and population (Acronym used: ART, antiretroviral therapy; RT-qPCR, real time quantitative PCR; NGS, next generation sequencing). * All percentages are calculated based on available data.

Sample	1700 (100)
Gender *	
Male, n (%)	1002 (74)
Female, n (%)	351 (26)
Risk factors for HIV infection *	
Vertical infection, n (%)	57 (9)
Sexual intercourse, n (%)	471 (72)
Injection drug use, n (%)	45 (7)
Blood transfusion, n (%)	84 (12)
Specimen types	
Plasma, n (%)	5 (38)
Serum, n (%)	4 (31)
Saliva, n (%)	1 (8)
Other, n (%)	3 (23)
Studies reporting patients on ART *	
Yes, n (%)	6 (60)
No, n (%)	4 (40)
TTV quantification method	
In house RT-qPCR, n (%)	9 (69)
Commercial RT-qPCR, n (%)	2 (15)
Metagenomic NGS, n (%)	1 (8)
In house end point qPCR, (%)	1 (8)

### 3.4. TTV Viral Load Kinetics in PLWH

All included studies investigated the potential relationship between TTV viral load and lymphocyte counts, aiming to determine whether fluctuations in TTV replication mirror changes in immune competence. However, only a limited number of studies reported absolute T-cell counts measured at the same time points as TTV viral load assessment.

Therefore, the present analysis focused primarily on the correlations already reported between TTV levels and lymphocyte parameters. Among studies included in the main circulating blood-based synthesis, TTV viral load was measured in plasma or serum and reported as log10 copies/mL. Plasma-based studies reported TTV viral loads ranging from 2.89 to 7.33 log10 copies/mL, while serum-based studies reported values ranging from 2.6 to 5.59 log10 copies/mL (Table 1). Studies using non-circulating or non-directly comparable matrices were excluded from this range. In particular, Thom et al. [28] reported TTV levels in bone marrow and spleen tissue, with higher values among subjects with AIDS than among pre-AIDS individuals. Similarly, Honorato et al. [25] evaluated TTV viral load in saliva and was therefore considered as salivary/mucosal evidence.

Abbate et al. [20] measured TTV levels in PBMCs and was interpreted as cellular compartment evidence. Finally, Fan et al. [29] reported TTV quantity using meta genomics next-generation sequencing as reads per million in blood, lower respiratory tract samples, and cerebrospinal fluid; therefore, these data were considered only as qualitative evidence.

All the studies that evaluated the association among TTV viral load and HIV viral load [25,27,31,32] found a direct correlation, indicating higher TTV viral load or abundance in individuals with increased HIV replication (Table 2). However, these studies differed in biological samples and quantification method. Overall, 10 studies (76.9%) [18,19,25,26,28,29,30,31,32,33] reported a significant inverse correlation between TTV viral load and CD4 cell count, with higher TTV levels observed in individuals with lower CD4 cell counts.

The association between TTV viral load and CD8 T cells was examined in only two studies (15.4%), yielding discordant findings [18,20]. One study reported a direct correlation with total CD8^+^ T-cell counts without further subset characterization [18], whereas the other identified differential associations across CD8 T-cell subsets [20].

Specifically, PBMC-associated TTV was inversely correlated with CD8^+^ central memory T cells and positively correlated with more differentiated subsets, including CD8 effector memory cells (r = 0.59, *p* < 0.002) and CD8^+^CD57^+^ cells (r = 0.464, *p* < 0.023).

This pattern suggests a shift toward terminally differentiated or senescent CD8 T-cell phenotypes, consistent with immune activation and/or chronic antigenic stimulation. Lastly, only two studies [18,29] examined the correlation between TTV viral load and the CD4/CD8 ratio. In these papers, a significant inverse correlation was consistently observed.

Statistical methods to measure association across the studies included Spearman correlation coefficients, Pearson R^2^ values, and chi-squared statistics. Therefore, they were re-ported descriptively only, according to the original studies and no pooled or comparative effect-size analysis was performed.

### 3.5. TTV Viral Load and ART

The duration of ART varied substantially across studies, from untreated individuals to patients with several years of ART (ranging from 12 to 65 months). Studies including ART-naïve PLWH or individuals with limited ART exposure reported markedly higher median TTV viral load, whereas lower TTV levels were generally observed among PLWH receiving long-term ART. Longitudinal analyses suggested a progressive decline in TTV viral load with increasing duration of ART, although considerable variability persisted. The study by Abate et al. [20], which enrolled subjects with acute HIV infection starting ART early, reported a transient increase in PBMC-associated TTV after three months of ART followed by a subsequent decline. By comparison, the study by Madsen et al. [32], which included chronically infected patients initiating ART, found a significant decrease in serum TTV viral load after 3 to 5 months of treatment. Taken together, these studies suggest that ART-associated changes in TTV levels may vary according to the phase of HIV infection and the biological compartment assessed.

## 4. Discussion

Despite marked methodological and clinical heterogeneity, this systematic review shows an inverse association between TTV viral load and CD4 cell count, supporting its potential role as a surrogate marker of immune competence in PLWH. Higher TTV levels were observed in ART-naïve individuals and in those with advanced immunosuppression, while longitudinal data suggest a decline with effective and sustained ART. A direct association with HIV viral load was observed when assessed, and emerging evidence suggests a relationship with CD8 cell activation and differentiation, although these findings remain limited. The inverse correlation with the CD4/CD8 ratio further supports the link between TTV replication and global immune impairment. Overall, these findings suggest that TTV viral load may reflect the degree of immune dysfunction beyond the CD4 cell count, although its clinical applicability requires cautious interpretation.

The evolution of HIV infection from a fatal disease [34,35] to a chronic manageable condition has been driven by advances in ART [36], resulting in substantial reductions in mortality and increased life expectancy [37]. However, despite these improvements, immune dysregulation, immunosenescence and chronic inflammation persist in PLWH, contributing to non-AIDS-related comorbidities [38,39]. As the HIV population ages, in the contemporary era, the most common causes of death include non-AIDS, non-hepatitis malignancies (13.7%), cardiovascular disease (8.3%), and liver-related conditions [35,40]. However, despite these advances in treatment and outcomes, the core biomarkers used in HIV monitoring have remained largely unchanged. CD4 cell count has long served as the principal immunological marker, providing critical prognostic information regarding disease progression and survival. It remains essential for guiding the starting/stopping of prophylaxis against opportunistic infections and for assessing immune recovery following ART initiation [41,42]. The introduction of plasma HIV-RNA quantification (viral load) in the mid-1990s represented a major milestone in HIV care and continues to serve as the primary marker of treatment response. Sustained virologic suppression, defined as <50 copies/mL according to the European AIDS Clinical Society guidelines, is the central goal of therapy [42]. However, CD4 cell count and HIV viral load, while essential, may not fully capture the complexity of immune recovery, immunosenescence, residual immune activation and impaired immunity [38].

TTV, a ubiquitous and non-pathogenic virus, has emerged as a potential indicator of immune function. Higher TTV viral load has been associated with impaired immune status across different clinical settings [19]. Observational studies report a high prevalence of TTV in both PLWH and healthy controls (approximately 96–99% vs. 91%, respectively), with significantly higher TTV viral loads observed in PLWH [25,27]. Elevated TTV levels are associated with lower CD4 counts and more advanced immunologic stages according to CDC classification [25,27]. A recently published systematic review focused on the kinetics of TTV viral load in hematopoietic stem cell transplantation recipients, showing a consensus among all the included studies about the kinetics of TTV viral load: TTV-DNA decreased dramatically after conditioning therapy, reaching the lowest levels around the time of hematopoietic engraftment, while the TTV viremia steadily increased, reaching peak levels at day +90 and +120 in most of the analyzed studies [43]. Moreover, TTV has been investigated as a predictor of immune recovery following ART initiation. In treatment-naïve individuals, baseline TTV plasma levels, when combined with CD4 count, were significantly associated with the magnitude of CD4 recovery during the first year of therapy (*p* = 0.004) [33]. PLWH with suboptimal immune reconstitution (<50 cells/µL increase) exhibited higher baseline TTV levels compared with those achieving robust recovery (>200 cells/µL increase), with mean values of 5.68 versus 4.99 log copies/mL (*p* = 0.011) [33].

The observed direct association among TTV-DNA and HIV viral load together with CD8 cell count suggests that TTV levels may also reflect ongoing immune activation [18]. The immune phenotyping data reported by Abbate et al. [20] further supports this interpretation and suggests a more complex relationship between TTV and immune status. Although PBMC-associated TTV was not correlated with CD4 T-cell count, it was inversely associated with CD8 central memory T cells and positively associated with more differentiated CD8 subsets, including CD8 effector memory cells (r = 0.59, *p* < 0.002) and CD8^+^CD57^+^ cells (r = 0.464, *p* < 0.023). CD8^+^CD57^+^ cells are commonly considered a marker of senescence and chronic antigenic stimulation. This observation is important because it extends the biological interpretation of TTV in PLWH, suggesting that TTV dynamics may also be linked to qualitative immune remodeling, particularly within the CD8 T-cell compartment. In populations characterized by acute infection, early ART, persistent immune activation, or immune aging, TTV may therefore capture aspects of immune dysfunction that are not fully reflected by absolute CD4 T cell count. Similar findings have been reported in other clinical contexts characterized by inflammation as a primary driver. In elderly populations, TTV load and species diversity have been associated with systemic inflammation, immune activation, and immunosenescence [44,45,46]. TTV viral load has also been investigated as a prognostic biomarker in autoimmune diseases [47]. In critically ill patients, it may serve as an indicator of susceptibility to opportunistic infections [48] while in respiratory viral infections it reflects both systemic and local immune activation and has been proposed as a noninvasive marker of immune dysregulation and thrombo-inflammatory risk [49].

This review has several limitations. First, the evidence is predominantly derived from observational studies, with a limited number of prospective and no randomized controlled trials, reducing the strength of causal inference. Second, substantial heterogeneity was observed in study design, populations, ART exposure, biological specimens, and TTV quantification methods, which likely contributed to variability in reported viral loads and precluded meta-analysis. Third, the interpretation of immunological associations is limited by the incomplete adjustment for clinical and biological variables across studies. Factors related to treatment history, demographic characteristics, concomitant viral infections, inflammatory background, and immune activation might influence TTV kinetics independently of HIV-related immune impairment, thereby contributing to inconsistent findings. This is particularly relevant in the study of Lapa et al. [24], which included patients with HIV/HCV coinfection and HCV-mono-infected controls during direct-acting antiviral therapy. In this setting, HCV infection and antiviral-induced clearance might lead to an alteration of host immune activation and TTV dynamics, potentially weakening the relationship between TTV viral load and CD4 T-cell count, therefore explaining the observed absence of association. Besides HCV coinfection, another relevant limitation is that only a limited number of included studies [29,33] assessed coinfection with other ubiquitous viruses, including herpesviruses. CMV, EBV, and HHV8 were evaluated in selected cohorts, while HSV and other herpesviruses were not systematically investigated. Therefore, the interaction between TTV replication, herpesvirus coinfections, and immune dysfunction in PLWH is still scarcely defined. Fourth, several clinically relevant associations (e.g., with CD8 T-cell subsets, CD4/CD8 ratio, and HIV viral load) were inconsistently assessed across studies, limiting the robustness of these findings. Fifth, most studies included adult populations and were conducted in Europe, potentially limiting generalizability. A further limitation is that PROSPERO registration was completed shortly after the literature search had been performed, rather than prospectively before study initiation. Finally, no universally validated TTV threshold has yet been established to predict immunosuppression or immune recovery better than CD4 T-cell count. The proposed cut-offs were study-specific and differed according to assay, population, and sample type. Thus, TTV viral load should currently be interpreted as a complementary marker of immune status rather than as a substitute for established immunological parameters. Then, the relationship between TTV replication and functional T-cell competence remains insufficiently defined because no direct functional assay of T-cell activity was performed in the included studies. However, this review provides a comprehensive and updated synthesis of the available evidence on TTV viral load in PLWH, including diverse populations and clinical settings. It highlights consistent immunological associations across heterogeneous studies and integrates both cross-sectional and longitudinal data. Importantly, it identifies key gaps in the literature, including the need for standardized methodologies and prospective studies, thereby providing a framework for future research on TTV as a biomarker of immune function.

Despite these observations, the clinical utility of TTV as a biomarker of immune function remains investigational across multiple settings, including HIV infection. Although it may provide complementary insights into immune status, its role in routine HIV monitoring is not yet established. Further well-designed, clinically validated studies—incorporating standardized patient populations, sampling time points, specimen types, and appropriate control groups—are needed to clarify its utility. In addition, harmonization of quantification methods and the definition of clinically meaningful thresholds are essential prerequisites for its implementation in clinical practice.

## Data Availability

No new data were created or analyzed in this study.

## Supplementary Materials

The following supporting information can be downloaded at: https://www.mdpi.com/article/10.3390/microorganisms14061386/s1, Prisma Checklist.
